# Small molecule natural products in human nasal/oral microbiota

**DOI:** 10.1093/jimb/kuab010

**Published:** 2021-02-06

**Authors:** Colin Charles Barber, Wenjun Zhang

**Affiliations:** Department of Plant and Microbial Biology, University of California, Berkeley, Berkeley 94720, USA; Department of Chemical and Biomolecular Engineering, University of California, Berkeley, Berkeley 94720, USA; Chan-Zuckerberg Biohub, San Francisco 94158, USA

**Keywords:** Human microbiome, Oral biology, Secondary metabolites, Biosynthesis

## Abstract

Small molecule natural products are a chemically diverse class of biomolecules that fulfill myriad biological functions, including autoregulation, communication with microbial neighbors and the host, interference competition, nutrient acquisition, and resistance to oxidative stress. Human commensal bacteria are increasingly recognized as a potential source of new natural products, which may provide insight into the molecular ecology of many different human body sites as well as novel scaffolds for therapeutic development. Here, we review the scientific literature on natural products derived from residents of the human nasal/oral cavity, discuss their discovery, biosynthesis, and ecological roles, and identify key questions in the study of these compounds.

## Introduction

Small molecule natural products are metabolites that often mediate critical biological and ecological functions, but without direct participation in metabolic processes essential for host growth and reproduction. Although natural products research has historically been biased toward ‘gifted’ producers like soilborne Actinobacteria and filamentous fungi, the microbiota of the human body is increasingly recognized as a source of potentially useful, bioactive secondary metabolites (Donia & Fischbach, 2015; Mousa et al., 2017; Milshteyn et al., 2018; Aleti et al., 2019; Hu & Zhang, 2020). Leveraging the human microbiome for natural products discovery is rooted in the logic that such metabolites are likely to comprise scaffolds relevant to human health, as factors in the chemical ecology of the microbiota, or as launchpads for pharmaceutical development.

The human body is a diverse ecological landscape; different body sites harbor vastly divergent microbial communities based on abiotic factors like water activity, pH, and nutrient availability, and biotic factors like immune system activity (Dewhirst et al., 2010; Ding & Schloss, 2014; Lloyd-Price et al., 2017; Proctor & Relman, 2017). Human microbiota-derived small molecules have been diligently reviewed (Donia & Fischbach, 2015; Mousa et al., 2017), but both producers and metabolites may be body site-specific, motivating a body site framework for discussing natural products from the human microbiome.

To focus our review, we choose the human nasal/oral cavity, which harbors a diverse, well-regulated assemblage of bacteria and some fungi that plays key roles in health and disease (Dewhirst et al., 2010; Gao et al., 2018; Jia et al., 2018). We restrict our taxonomic focus to strains identified in the Human Oral Microbiome Database, which lists over 600 bacterial species found in the human nasal/oral cavity (Chen et al., 2010; Dewhirst et al., 2010), and microbial species known to inhabit the nasal/oral cavity. With the exception of *S. aureus*, a well-described nasal inhabitant (Sakr et al., 2018; Wertheim et al., 2005), we omit discussion of secondary metabolites produced by ESKAPE pathogens, a group of six highly virulent nosocomial pathogens whose small molecule natural products have been reviewed elsewhere (Carroll & Moore, 2018; Gulick, 2017). Additionally, we do not review the wealth of literature on ribosomally synthesized and post-translationally modified natural products (RiPPs) originating from human oral bacteria; many such RiPPs are also reviewed elsewhere (Jakubovics et al., 2014; Merritt & Qi, 2012; Zheng et al., 2015). Where possible, we discuss the genomic origins, biosynthesis, and biological functions of each small molecule natural product and identify unanswered questions relevant to oral biology. Greater detail is offered for recent examples of nasal/oral microbiota-derived natural products.

Although only a handful of nasal/oral microbiota-derived natural products have been isolated and characterized, many possess useful antimicrobial, probiotic, and immunomodulatory properties. Genomic and metabolomic data indicate the nasal/oral microbiome is a treasure chest of undiscovered small molecules (Aleti et al., 2019; Edlund et al., 2017), portending exciting advances in nasal/oral microbiota-derived natural products.

## Mutanobactins

**Figure 1.**
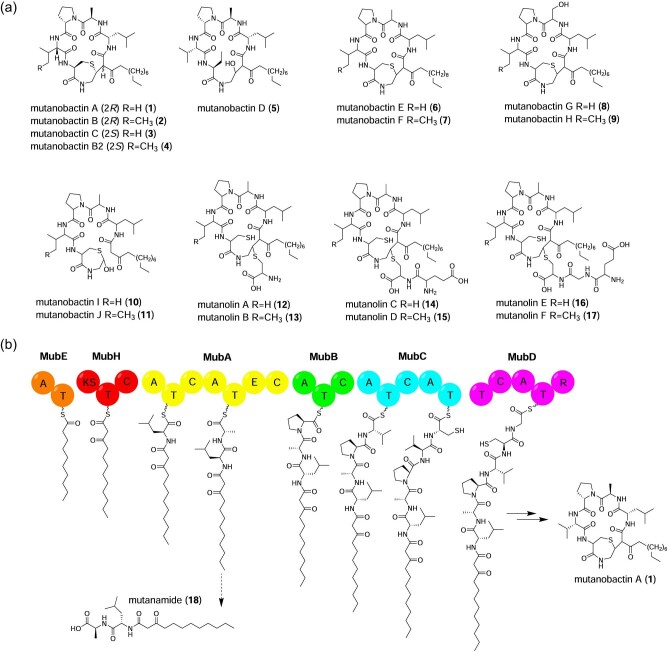
(a) Proposed mutanobactin A (**1**) NRPS assembly line, including derailment event to produce mutanamide (**18**). Domain abbreviations: A, adenylation; C, condensation; E, epimerization; KS, ketosynthase; R, reduction; T, thiolation. (b) Structures for mutanobactin congeners, including the mutanolins. Note that stereochemistry has not been resolved for mutanobactins E–J and mutanolins A–F.

The mutanobactin family of non-ribosomal lipopeptides, produced by *Streptococcus mutans*, is one of the best-studied compound families arising from the human oral microbiota. *S. mutans* is a facultative anaerobe ubiquitous in the human nasal/oral cavity and is recognized as a major driver of dental caries (Bowen et al., 2018; Loesche, 1986). Ajdić et al. identified several insertion elements in the *S. mutans* UA159 genome, including TnSmu2, a genomic island containing several NRPS-encoding genes (Ajdić et al., 2002; Wu et al., 2010). This gene cluster, which is also present in *S. mutans* UA140 and MT4653, was targeted for product elucidation via gene deletion (Wu et al., 2010). Although wild-type colonies of UA140 and M4653 are yellow, deletion of the first NRPS gene resulted in white colonies, and HPLC profiling confirmed that production of the hypothetical molecule was abrogated in the deletion mutants (Wu et al., 2010). Wild-type UA159 is white colored, but deletion of the first NRPS gene also eliminated production (Wu et al., 2010). The hypothetical molecule, named mutanobactin A (**1**), was then purified from UA159 and structurally characterized (Joyner et al., 2010). Mutanobactin A is a hybrid NRPS/PKS metabolite with an unusual 1,4-thiazepan-5-one ring system and a C10 fatty acyl tail (Joyner et al., 2010) (Fig. 1a). The structures of mutanobactins B, C, and D (**2, 3, 5**), congeners of mutanobactin A, were elucidated through a robust combination of HRMS as well as several NMR experiments (Wang et al., 2012) (Fig. 1a). Zvanych et al. identified and proposed partial structures for a litany of mutanobactin-like molecules through MS/MS fragmentation and isotope labeling studies. First, Zvanych et al. found several other mutanobactin congeners, B2 (**4**) and E–J (**6–11**) (Zvanych et al., 2015) (Fig. 1a). Six other structures, the mutanolins (**12–17**), a consequence of cysteine incorporation in the final module of the NRPS assembly line, were also found (Zvanych et al., 2015) (Fig. 1a). Lastly, mutanamide (**18**), a biosynthetic derailment product, was identified using the chemoinformatic search algorithm iSNAP (Ibrahim et al., 2012; Zvanych et al., 2015) (Fig. 1a). The mutanamide structure was solved through MS/MS, Marfey's method, and NMR (Zvanych et al., 2015).

Mutanobactin biosynthesis proceeds through a hybrid NRPS/PKS assembly line pathway. Biosynthesis is initiated by loading decanoic acid onto the MubE starter module, which is then elongated by incorporation of a malonyl-CoA extender unit promoted by a PKS module and various amino acids by six NRPS modules (Wang et al., 2020; Zvanych et al., 2015) (Fig. 1b). Structural diversity arises from promiscuous amino acid incorporation on the NRPS assembly line (Wang et al., 2020; Zvanych et al., 2015) (Fig. 1b). The nascent polypeptide is then released by the terminal reductase domain of MubD as an aldehyde, which can undergo macrocyclization through an aldol addition (Wang et al., 2020). The 1,4-thiazepane ring is formed through spontaneous C–S bond formation through nucleophilic attack by the thiol of the cysteinyl moiety on the hydroxylated carbon backbone resulting from the previous aldol addition (Wang et al., 2020). These cascading nonenzymatic reactions are the source for mutanobactin structural diversity not explained by the NRPS assembly line (Wang et al., 2020; Zvanych et al., 2015).

The mutanobactins are thought to carry out several functions. First, mutanobactins aid in oxidative stress resistance. Deletion of the *mub* locus in UA140, UA159, and MT4653 results in lower growth rate and stationary phase OD in aerobic conditions (Wu et al., 2010). Challenging both the wild-type and deletion mutants with H_2_O_2_ greatly reduces growth rate under anaerobic conditions (Wu et al., 2010). Interestingly, *mub* deletion prevents mature biofilm formation under aerobic but not anaerobic conditions, suggesting a link between mutanobactin-mediated ROS resistance and biofilm formation (Wu et al., 2010). Mutanobactin-mediated ROS tolerance is likely relevant to *S. mutans* survival in an oral context, where *S. mutans* must successfully grow in both aerobic and anaerobic conditions, survive ROS challenge by microbial competitors and the host, and form biofilms on tooth surfaces (Lemos et al., 2019). However, lack of chemical complementation data weakens these results. The biological importance of mutanobactin-mediated ROS tolerance *in situ* also remains underexplored.

Second, some mutanobactins regulate the yeast-mycelium transition in the opportunistic oral pathogen *Candida albicans*. This phenomenon was first observed during microscopic studies in which hyphal formation in *C. albicans* was observed in the presence of *S. mutans* UA159 Δ*mub* but not wild-type *S. mutans* UA159 (Joyner et al., 2010). The role of mutanobactin A was confirmed by treating *C. albicans* with purified mutanobactin A in the absence of *S. mutans* producers, which inhibited *C. albicans* filamentation (Joyner et al., 2010). As biofilm formation depends on filamentation (Brown et al., 2007; Grow et al., 2002; Saville et al., 2003; Wang et al., 2012), mutanobactins A, B, and D all efficiently (<100 μM) inhibited *C. albicans* biofilm formation in an *in vitro* assay, with mutanobactin D having the lowest IC_50_ at 5.3 ± 0.9 μM (Wang et al., 2012). These three mutanobactins all inhibit biofilm formation more efficiently than farnesol, discussed later in this review (Wang et al., 2012). Mutanamide is more efficacious than mutanobactin A or B, inhibiting hyphal formation more than mutanobactin A or B at 10, 100, and 1 mM (Zvanych et al., 2015). Importantly, mutanobactins do not reduce *C. albicans* viability, suggesting the molecular mechanism underlying biofilm inhibition is specific to the biofilm pathway (Wang et al., 2012). The mutanobactins are therefore part of a growing number of human microbiome-derived metabolites involved in interkingdom communication (Aleti et al., 2019; Donia and Fischbach, 2015; Hu & Zhang, 2020). These findings imply that mutanobactins might attenuate *C. albicans* virulence because filamentation and biofilm formation are required for pathogenicity (Brown et al., 2007; Grow et al., 2002; Joyner et al., 2010; Saville et al., 2003; Wang et al., 2012) Indeed, Barbosa and coworkers found that *S. mutans* culture filtrate blunts hyphal formation and attenuates *C. albicans* virulence in a *Galleria mellonella* infection model, although a direct link to mutanobactin production has not been firmly established in any *in vivo* model (Barbosa et al., 2016). Additional research is needed to establish the biological roles of mutanobactin in *C. albicans* development and pathogenicity in an oral context.

Finally, the mutanobactins have immunomodulatory properties. In LPS-stimulated RAW264.7 macrophages, mutanobactin B upregulates the pro-inflammatory cytokines IL-6 and IL-12, but downregulates three others, namely MCP-1, G-CSF, and TNF-α (Zvanych et al., 2015). The immunomodulatory effects of the also-tested mutanamide and mutanobactin A were less obvious (Zvanych et al., 2015). The mutanobactins are therefore one of a growing number of compound families associated with immunomodulation specifically and communication with the human host generally (Aleti et al., 2019; Donia and Fischbach, 2015; Hu & Zhang, 2020; Milshteyn et al., 2018; Mousa et al., 2017), but further research is required to understand how various mutanobactin congeners modulate the oral immune response and the impact of mutanobactin-induced immune response on *S. mutans* in the nasal/oral context.

## Lugdunin

**Figure 2.**
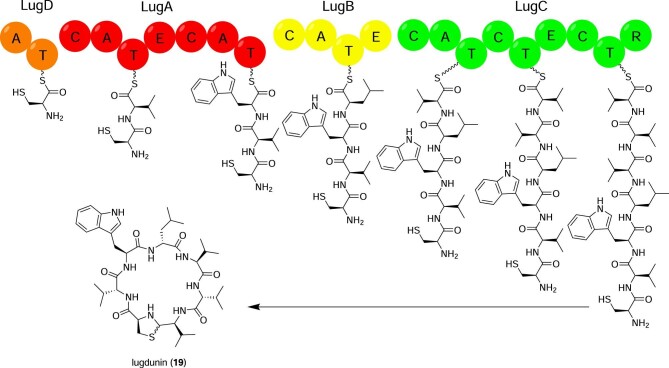
Proposed lugdunin (**19**) biosynthetic pathway. Domain abbreviations: A, adenylation; C, condensation; E, epimerization; R, reduction; T. thiolation.

*Staphylococcus lugdunensis* is a nasal/oral commensal and the only known producer of lugdunin (**19**) (Fig. 2), a cyclic non-ribosomal peptide with potent antimicrobial activity. Its producer, *S. lugdunensis* IVK28, was initially identified in a bioactivity screening against the opportunistic pathogen *Staphylococcus aureus* USA300, and an untargeted transposon mutagenesis strategy identified an NRPS-encoding genetic locus as the source of the hypothetical antimicrobial (Zipperer et al., 2016). Engineering the putative *lug* locus with the strong *xylAB* promoter and a corresponding increase in antimicrobial activity in the engineered strain over the wild-type confirmed the candidacy of the *lug* locus as the source of the antimicrobial (Zipperer et al., 2016). A robust combination of NMR, HRMS, and Marfey's method was used to solve the structure of lugdunin, which has an unusual thiazolidine ring (Zipperer et al., 2016).

Biosynthesis of lugdunin proceeds mostly according to conventional NRPS logic. Unusually, LugC has only one A domain yet two T domains and three C domains, leading Zipperer et al. to propose that three consecutive valine moieties are incorporated by LugC (Zipperer et al., 2016) (Fig. 2). After chain release by the terminal reductase domain, the peptide backbone cyclizes through nucleophilic attack by the cysteinyl amine on the aldehyde, and the thiazolidine ring is closed by attack of the cysteinyl thiol on the resultant imine (Zipperer et al., 2016). Total synthesis was achieved for structural proof, and synthetic analogs have been synthesized for structure-activity relationship studies (Zipperer et al., 2016; Schilling et al., 2019).

Lugdunin possesses potent inhibitory activity (<10 μg/ml) against several Gram-positive bacteria, including methicillin-resistant *S. aureus*, vancomycin-resistant *Enterococcus* isolates, and *Listeria monocytogenes*, but not Gram-negatives (Zipperer et al., 2016). Lugdunin likely acts through disruption of the transmembrane pH gradient; inhibitory activity also requires the thiazolidine ring and alternating stereochemistry in the peptide backbone (Schilling et al., 2019). Remarkably, in a murine infection model, lugdunin successfully curtails *S. aureus* skin infection (Zipperer et al., 2016). Similarly, in a cotton rat model, *S. lugdunensis* IVK28 heavily reduces *S. aureus* colonization of the nasal cavity over a non-producing deletion mutant, and data from human nasal swabs indicate that nasal carriage of *S. aureus* is significantly lower in *S. lugdunensis* carriers than non-carriers (Zipperer et al., 2016). In addition to an impressive array of per se inhibitory activities *in vitro* and *in vivo*, lugdunin acts in concert with the immune system against *S. aureus*. In specific, lugdunin acts synergistically with the human antimicrobial peptides DCD-1(L) and LL-37 to kill MRSA, amplifies the innate immune response in primary human keratinocytes, and recruits phagocytes on skin in a murine model (Bitschar et al., 2019). Because *S. aureus* nasal carriage predisposes carriers to infection (Krismer et al., 2017), these data strongly support the potential for lugdunin as a launchpad for future therapeutic development or *S. lugdunensis* as a probiotic candidate, although additional work is needed for a more complete ecological picture of *S. lugdunensis* in the nasal/oral community.

## Reutericyclin and Mutanocyclin

**Figure 3.**
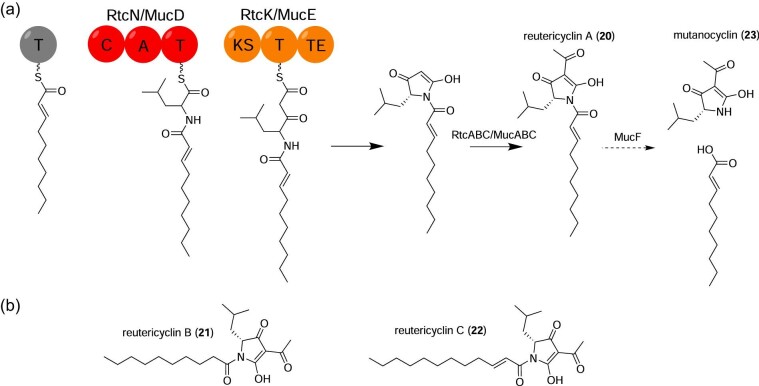
(a) Proposed reutericyclin A (**20**) biosynthetic pathway. Deacylation to produce mutanocyclin (**23**) is also shown. Domain abbreviations: A, adenylation; C, condensation; KS, ketosynthase; T, thiolation; TE, thioesterase. (b) Structures of the congeners reutericyclin B and C.

Although *Lactobacillus reuteri* is commonly known as a gut bacterium, *L. reuteri* has been detected in the human nasal/oral cavity and has been studied as an anticariogenic oral probiotic (Cagetti et al., 2013; Chen et al., 2010). Reutericyclin-family compounds are NRPS-produced tetramic acids first discovered in the fermentative Gram-positive *L. reuteri* LTH2584 (Gänzle, 2004; Gänzle et al., 2000). Reutericyclin A (**20**) was targeted for purification after it was found that *L. reuteri* LTH2584 produced a putative non-RiPP small molecule with inhibitory activity against *Lactobacillus sanfranciscensis* ATCC 27 651, the first indicator strain tested, as well as other Gram-positive bacteria like *Bacillus subtilis* and *S. aureus*. (Gänzle et al., 1995, 2000) Bioactivity-guided fractionation identified only one active compound, confirmed by ESI-MS, and NMR and methanolysis enabled elucidation of the reutericyclin A structure (Gänzle et al., 2000; Höltzel et al., 2000). Reutericyclin A possesses an *N*-linked α,β-unsaturated fatty acyl tail, unprecedented for tetramic acid compounds from nature (Gänzle et al., 1995, 2000) (Fig. 3a). Total synthesis of reutericyclin was achieved via Dieckmann condensation (Böhme et al., 2005).

The set of *L. reuteri* strains known to produce reutericyclin A was expanded to include the industrial sourdough fermentation strains *L. reuteri* TMW1.106, TMW1.112, and TMW1.656 based on bioactivity and HPLC confirmation of reutericyclin production (Gänzle & Vogel, 2003). Yet until recently, the genomic origins of reutericyclin biosynthesis eluded researchers (Gänzle & Vogel, 2003; Lin et al., 2015). Genome comparison between reutericyclin-producing *L. reuteri* and non-producing *L. reuteri* facilitated identification of a candidate hybrid NRPS/PKS biosynthetic gene cluster, named *rtc* (Lin et al., 2015). Deletion of the *rtc* core biosynthetic genes abrogated production of reutericyclin A, confirming its role in reutericyclin A production (Lin et al., 2015).

The reutericyclin biosynthetic pathway was proposed mostly using thiotemplated assembly logic of the core NRPS/PKS proteins. Although the reutericyclin NRPS/PKS machinery lacks an epimerization domain, the reutericyclin scaffold features a d-leucyl subunit, which was initially proposed to result from incorporation of d-leucine by RtcN (Lin et al., 2015). Production of d-leucine by a promiscuous isoleucine 2-epimerase has been observed in *Lactobacillus* spp. (Mutaguchi, Ohmori, Akano, et al., 2013; Mutaguchi, Ohmori, Wakamatsu, et al., 2013) However, incorporation of exogenously supplied, isotope-labeled l-leucine but not d-leucine supports the existence of an unknown epimerase which acts on the NRPS assembly line (Tang et al., 2020). The precise timing of the proposed epimerization is not currently known (Tang et al., 2020). RtcK extends the growing chain by two carbons according to canonical decarboxylative condensation of malonyl-CoA, and the TE domain of RtcN catalyzes cyclization to form the five-membered ring (Lin et al., 2015) (Fig. 3a). Finally, RtcABC acetylates the cyclized product to yield **20** (Lin et al., 2015) (Fig. 3a). The RtcABC system is homologous to PhlABC, a multiprotein ensemble that acetylates phloroglucinol to 2,4-diacetylpholorglucinol, an antimicrobial and chemical messenger produced by some pseudomonads (Bangera & Thomashow, 1999; Clifford et al., 2016; Lin et al., 2015). Resistance to reutericyclin is likely conferred by the transporter RtcT; *rtcT* deletion is only possible in a non-producing Δ*rtcN* mutant (Lin et al., 2015). *L. reuteri* Δ*rtcN* Δ*rtcT* is also sensitive to exogenously supplied reutericyclin (Lin et al., 2015). Deletion of the attendant regulatory genes *rtcRS* is also permitted only in a nonproducing strain, suggesting *rtcRS* regulate the resistance phenotype (Lin et al., 2015).

Discovery of the complete reutericyclin BGC facilitated discovery of reutericyclin A-like compounds in other oral residents. Liu et al. identified 22 biosynthetic gene clusters in the oral cariogen *S. mutans* homologous to the reutericyclin BGC with conserved domain order in the putative core NRPS/PKS biosynthetic proteins (Liu et al., 2016). Using a newly developed *S. mutans* heterologous expression platform for small molecule natural products from anaerobic bacteria, Hao et al. expressed a representative *rtc*-like BGC, which yielded mutanocyclin (**23**), a reutericyclin A congener without an *N*-acyl tail (Hao et al., 2019) (Fig. 3a). Additional reutericyclin congeners, reutericyclins B (**21**) and C (**22**), resulting from variations in fatty acyl chain length and saturation, were discovered in *S. mutans* B04Sm5; mutanocyclin production was also reported in this strain (Tang et al., 2020) (Fig. 3b). Analogous to reutericyclin biosynthesis in *L. reuteri*, a three-protein ensemble, MucABC, is proposed to acetylate the reutericyclin scaffold (Tang et al., 2020). MucF was proposed to be the deacylase responsible for production of **23** from the reutericyclin scaffold (Tang et al., 2020) (Fig. 3a). Interestingly, growth of *S. mutans* B04sm5 Δ*mucF* is heavily impaired relative to the wild-type and Δ*mucD* strains, suggesting MucF detoxifies mutanocyclin pre-products (Tang et al., 2020).

Reutericyclin A possesses potent bioactivity, often in the sub-μg/ml range, against many Gram-positive bacteria, including *Staphylococci, Lactobacilli*, and *Bacilli* (Gänzle, 2004). Representatives of these genera are frequently present in the human nasal/oral cavity (Chen et al., 2010; Jia et al., 2018). No fungi nor Gram-negative bacteria are known to be susceptible to reutericyclin A (Gänzle, 2004). Activity studies confirm that reutericyclin A dissipates transmembrane pH but does not impact transmembrane gradients of other ions, suggesting reutericyclin is a proton-ionophore (Gänzle & Vogel, 2003). Reutericyclin A therefore joins a swelling family of tetramic acid ionophores with activity against Gram-positive targets (Klapper et al., 2019). Perhaps due to reutericyclin production, reutericyclin-producing *L. reuteri* are known to modulate fecal microbial community composition in animal models, but additional work is needed to evaluate the effect of reutericyclin on microbial communities where reutericyclin producers reside (Yang et al., 2015; Zhao et al., 2019).

On the other hand, mutanocyclin is not known to have any antibacterial activity, including against Gram-positive competitors and oral commensals like *Streptococcus sanguinis* and *Streptococcus gordonii* (Hao et al., 2019). But mutanocyclin suppresses CD45 + leukocyte infiltration in a Matrigel plug assay, suggesting an anti-inflammatory role for mutanocyclin (Hao et al., 2019). Additional research is needed to understand the host immune response to mutanocyclin.

## Mutanofactin

**Figure 4.**
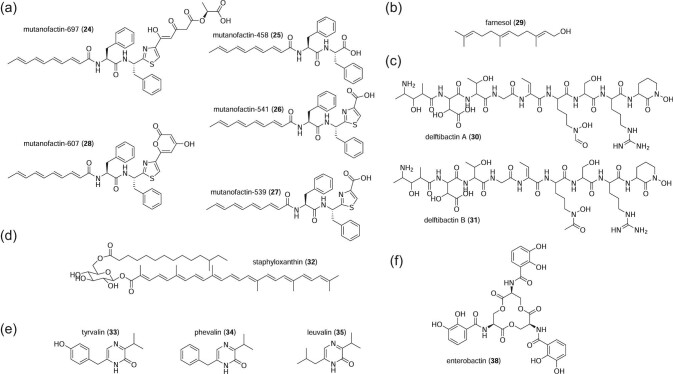
(a–g) Structures of metabolites discussed.

The oral cariogen *S. mutans* is also the producer of mutanofactin-697 (**24**), which facilitates biofilm formation (Li et al., in press). Bioinformatic analysis of 57 *S. mutans* strains, including clinical isolates, revealed several *S. mutans* groups distinguished by secondary metabolite BGCs (Li et al., in press). Intriguingly, Group I *S. mutans*, the only strains in the panel to harbor BGC1 and BGC2, also demonstrate strong biofilm formation (Li et al., in press), a critical process in the development of dental caries (Kuramitsu et al., 2007; Lamont et al., 2018). Disruption of BGC1 but not BGC2 diminishes biofilm formation on a polystyrene surface as well as an acrylic tooth model (Hahnel et al., 2008; Li et al., in press). The structures of the major product of BGC1 (hereafter *muf*), mutanofactin-697, as well as four minor products (**25–28**), were proposed on the basis of multiple NMR experiments and HRMS (Li et al., in press) (Fig. 4a). Biosynthesis of mutanofactin-697 is proposed to follow conventional NRPS/PKS assembly line logic, with the exception of chain release resulting from terminal C domain-mediated esterification between l-lactic acid and the nascent NRP/PK chain (Li et al., in press). Minor products are proposed to be consequences of assembly line derailments (**25–27**) or spontaneous cyclization before or after chain release (**28**) (Li et al., in press) (Fig. 4a).

Critically, complementation of Δ*muf S. mutans* with mutanofactin-697 rescues biofilm formation. Chemical complementation also restores cell surface hydrophobicity, suggesting a physicochemical mechanism for adhesion promoted by mutanofactin-697. Exogenously supplied mutanofactin-697 also promotes biofilm formation and increases cell surface hydrophobicity in *muf*^–^ *S. mutans*, implying that nonproducing *S. mutans* might benefit from mutanofactin-697 *in situ* (Li et al., in press). Additional research is needed to elucidate the role of mutanofactin-697 in dental caries progression.

## Farnesol

Farnesol (**29**) is a product of the dimorphic fungus and causative agent of oral thrush *C. albicans* with myriad activities. The structure of farnesol, a sesquiterpene intermediate in the sterol biosynthesis pathway, was known long before its bioactivity was uncovered, and its biosynthetic origins were confirmed by inhibiting squalene synthase in *C. albicans* (Hornby et al., 2003; Polke et al., 2018) (Fig. 4b). Hornby et al. identified farnesol as the molecular driver of quorum sensing in *C. albicans*; indeed, it was the first known quorum sensing metabolite in a eukaryotic system (Hornby et al., 2001). Farnesol regulates the yeast-mycelium transition by promoting yeast-like growth at high concentration, corresponding to high cell density (Hornby et al., 2001). Later work demonstrated that farnesol can also induce yeast formation in preformed hyphal cells (Lindsay et al., 2012; Polke et al., 2018). By inhibiting filamentation, farnesol also inhibits biofilm formation in *C. albicans* (Ramage et al., 2002). Farnesol therefore plays a significant role in *C. albicans* pathogenesis and virulence, which depends on successful biofilm development (Chandra et al., 2001; Navaranthna et al., 2007; Wall et al., 2019). The molecular mechanism underlying farnesol-mediated autoregulation is reviewed in detail elsewhere (Gupta et al., 2018; Polke et al., 2018).

## Delftibactin

*Delftia acidovorans* is a Betaproteobacterium positively associated with oral health and rare non-oral opportunistic pathogen (Chen et al., 2015; Kawamura et al., 2011; Mahmood et al., 2012). *D. acidovorans* is also known to dominate gold nugget microbiota, although soluble gold is toxic to microbial life (Reith et al., 2010; Johnston et al., 2013). In contrast to the gold nanoparticle-accumulating bacterium *Cupriavidus metallidurans, D. acidovorans* was found to secrete a diffusible metabolite that sequesters extracellular soluble Au(III) (Johnston et al., 2013). Knocking out *delG*, which was predicted to code for an NRPS, abrogates production of a non-ribosomal peptide metabolite, delftibactin A (**30**), and causes sensitivity to soluble gold (Johnston et al., 2013) (Fig. 4c). Delftibactin A co-precipitates with gold and restores gold resistance to the deletion strain, confirming the unprecedented ability of delftibactin A to confer resistance to soluble gold (Johnston et al., 2013). Delftibactin A can also chelate other + 3 cations including iron and gallium, suggesting a role as a multipurpose chelator (Johnston et al., 2013). The congener Delftibactin B (**31**) is distinguished from delftibactin A by acetylation, rather than formylation, of the *N*^δ^*-*hydroxyornithine moiety (Johnston et al., 2013) (Fig. 4c). All delftibactin structures have been confirmed by NMR (Johnston et al., 2013).

Delftibactin also behaves as a potent (around 10 μg/ml) antimicrobial, especially against notorious Gram-positive pathogens like vancomycin-resistant *Enterococcus* and methicillin-resistant *S. aureus* (Tejman-Yarden et al., 2019). The *in situ* relevance of delftibactin-producing *D. acidovorans*, especially in microbiota associated with gold crowns, fillings, and plating, is unknown.

Delftibactin biosynthesis proceeds according to canonical NRPS logic. The delftibactin BGC encodes the requisite enzymes for β-hydroxyaspartate, *N*^δ^-hydroxyornithine, and *N*^δ^-formyl-*N*^δ^-hydroxyornithine functions common to siderophore biosynthesis. (Barry & Challis, 2009; Hider & Kong, 2010; Johnston et al., 2013) Interestingly, two other Proteobacteria harbor homologous gene clusters, suggesting a role for delftibactin beyond *D. acidovorans* (Aiman et al., 2018; Hong et al., 2017).

## Staphyloxanthin

Staphyloxanthin (**32**) is a carotenoid pigment first discovered in the opportunistic pathogen and well-known nasal commensal *S. aureus* (Armstrong, 1997; Laux et al., 2019) (Fig. 4d). *S. aureus*, so named for its color, has long been recognized as a potential source of pigment compounds; indeed, triterpenoid carotenoids are readily isolated from *S. aureus* (Armstrong, 1997). Staphyloxanthin biosynthesis requires two farnesyl precursors, which are condensed and oxidized in a stepwise fashion to form 4,4′-diaponeurosporenic acid (Pelz et al., 2005). Successive glycosyl and fatty acyl transfers yield the final product, notable for its extensive conjugated pi bond system (Miethke & Marahiel, 2007) (Fig. 4d).

Staphyloxanthin was hypothesized to promote oxidative stress resistance. Indeed, fitness of Δ*crt* mutants is greatly reduced relative to wild-type in a variety of oxidative challenge assays, even after controlling for catalase activity (Clauditz et al., 2006). Wild-type *S. aureus* also outperform Δ*crt* mutants in a neutrophil killing assay, suggesting that staphyloxanthin might aid *S. aureus* in evading the immune system, implying a role in *S. aureus* virulence (Clauditz et al., 2006; Liu et al., 2005). Staphyloxanthin biosynthetic genes have therefore attracted attention as a target for anti-virulence drug discovery (Maura et al., 2016), leading to investigation of several virulence-attenuating candidates (Liu et al., 2008; Lee et al., 2013; Leejae et al., 2013; Ribeiro et al., 2020; Song, Lin, et al., 2009; Song, Liu, et al., 2009).

## Aureusimine Pyrazinones

*S. aureus* also produces a class of non-ribosomal peptides with enigmatic biological roles. This family of non-ribosomal peptides was independently discovered by two groups (Wyatt et al., 2010; Zimmermann & Fischbach, 2010). Three aureusimine pyrazinone congeners, tyrvalin (**33**), phevalin (**34**), and leuvalin (**35**), were isolated following targeted knockout of *pznA/ausA*, which encodes a dimodular NRPS (Wyatt et al., 2010; Zimmermann & Fischbach, 2010). PznA/AusA contains a terminal reductase domain which carries out a two-electron reductive release to produce a dipeptide aldehyde that spontaneously cyclizes to form the pyrazinone (Wyatt et al., 2010; Zimmermann & Fischbach, 2010) (Fig. 4e). *In vitro* reconstitution of the aureusimine pyrazinone pathway supports this hypothesis (Wilson et al., 2013).

The second module of PznA promiscuously accepts both tyrosine and phenylalanine, explaining why phevalin and tyrvalin are produced 1:1 while leuvalin is a minor product in *S. aureus* (Zimmermann & Fischbach, 2010). *In vivo* and *in vitro* studies reveal a variety of substituted aryl rings that can be processed by AusA (Wyatt et al., 2012). Homologs of this gene cluster are present in other opportunistically pathogenic *Staphylococcus* spp., including *S. epidermidis, S. capitis*, and *S. lugdunensis*, but the major product varies; in *S. epidermidis*, for example, the major product is tyrvalin (Wyatt et al., 2010; Zimmermann & Fischbach, 2010).

The function of staphylococcal pyrazinones remains a topic of debate. None of the staphylococcal pyrazinones have detectable antibiotic activity (Zimmermann & Fischbach, 2010). Although the aureusimine pyrazinones were thought to regulate virulence (Wyatt et al., 2010), this phenotype was later revealed to be confounded by an unexpected mutation in a bona fide virulence regulator (Sun et al., 2010; Wyatt et al., 2011). Instead, the aureusimine pyrazinones might regulate a suite of metabolic genes involved in electron transfer processes (Wyatt et al., 2011). Although phevalin production is upregulated during biofilm development over planktonic growth, exogenous phevalin does not substantially influence *S. aureus* development (Secor et al., 2012). Aureusimine pyrazinones might play a role in host-microbe interactions. Regulation of multiple biological processes in host cells, including apoptosis, is influenced by phevalin (Secor et al., 2012). In a virulence factor screen, Blättner et al. found that the *ausAB* operon is required for phagosomal escape and induction of epithelial cell death (Blättner et al., 2016). In a murine model of pneumonia, an *ausB* knockout strain is outcompeted by the wild-type and *ausB* deletion partially ablates virulence, suggesting that aureusimine pyrazinones might be virulence factors in some *S. aureus* strains after all (Blättner et al., 2016). The discovery of a widespread class of dipeptide aldehydes produced by gut commensals (Guo et al., 2017) further complicates the question of aureusimine pyrazinone bioactivity. Although many of these dipeptide aldehydes spontaneously cyclize to their pyrazinone congener, the discovery that dipeptide aldehydes inhibit host proteases and that some might be stabilized under physiological conditions or by structural features like an *N*-acyl tail (Guo et al., 2017) raises fundamental questions about the active form of the aureusimine pyrazinones, their lifetime *in situ*, and their production conditions.

## Siderophores of *S. aureus*

**Figure 5.**
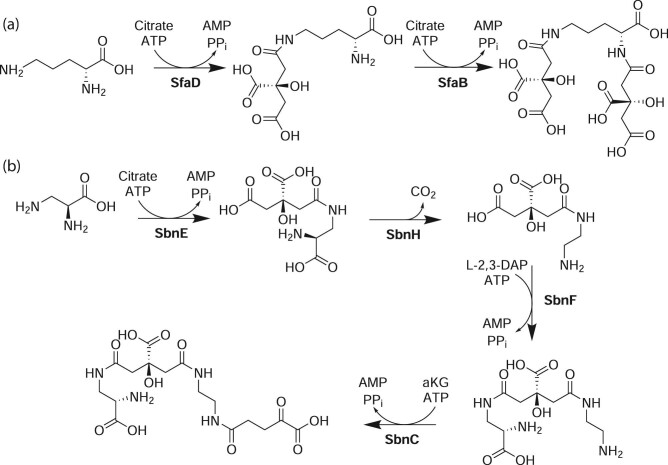
(a) Proposed staphyloferrin A (**36**) biosynthetic pathway. Adapted from Cotton et al. (2009). (b) Proposed staphyloferrin B (**37**) biosynthetic pathway. Adapted from Cheung et al. (2009).

Two siderophores have been isolated and characterized from *S. aureus*: staphyloferrin A (**36**) and B (**37**) (Cassat and Skaar, 2012; Cotton et al., 2009; Courcol et al., 1997; Dale, Doherty-Kirby, et al., 2004; Drechsel et al., 1993; Konetschny-Rapp et al., 1990. Staphyloferrin A and B were identified in *S. aureus* (A) or *Staphylococcus hyicus* (B) under iron starvation conditions, but *S. aureus* can produce both siderophores (Meiwes et al., 1990; Haag et al., 1994). Indeed, staphyloferrin A and B can be produced by many *Staphylococcus* spp., but staphyloferrin A is detectably produced by more strains than staphyloferrin B (Haag et al., 1994). Both are complexone-type siderophores but are structurally distinct (Haag et al., 1994; Meiwes et al., 1990) (Fig. 5a,b).

Heterologous expression and *in vitro* reconstitution confirm a two-step NRPS-independent siderophore (NIS) pathway to synthesize staphyloferrin A. SfaD condenses one molecule of citrate onto the δ-amine of d-ornithine, then SfaB condenses another citrate molecule onto the α-amine of the ornithine moiety to produce **36** (Cotton et al., 2009) (Fig. 5a). d-Ornithine is supplied by the PLP-dependent amino acid racemase, SfaC (Cotton et al., 2009). Staphyloferrin A uptake is driven by HtsABC, previously characterized as a heme transport system, in concert with FhuC, a transporter ATPase in the ferric hydroxamate uptake system (Beasley et al., 2009; Grigg et al., 2010).

Staphyloferrin B is likewise synthesized through an NIS pathway. *In vitro* reconstitution confirms that staphyloferrin B biosynthesis arises from citrate, α-ketoglutarate, and two l-2,3-diaminopropionate (DAP) molecules (Cheung et al., 2009; Dale, Sebulsky, et al., 2004) (Fig. 5b). SbnCEF form the minimal set of NIS synthetases in the staphyloferrin B biosynthetic pathway, while SbnH catalyzes PLP-dependent decarboxylation of citryl-DAP to produce citryl-diaminoethane for downstream processing (Cheung et al., 2009). Staphyloferrin B uptake is driven by the SirABC system in tandem with the FhuC transporter ATPase (Dale, Sebulsky, et al., 2004; Speziali et al., 2006). Consistent with this observation, staphyloferrin B production is controlled by a heme-responsive regulator (Laakso et al., 2016).

Iron assimilation, often mediated by siderophores, is a requirement for virulence in several pathogens (Konetschny-Rapp et al., 1990; Miethke & Marahiel, 2007), making staphyloferrin a target of virulence studies. Staphyloferrin A and B can sequester iron from human holo-transferrin, suggesting a role for the staphyloferrins in *S. aureus* virulence (Park et al., 2005; Beasley et al., 2011). In a rat model of infective endocarditis, deletion of *sbn* locus attenuates virulence (Hanses et al., 2014). Staphyloferrin B production is similarly linked to virulence in a murine kidney abscess model (Dale, Doherty-Kirby, et al., 2004). Staphyloferrin A and B can functionally complement each other, but deletion of both the *sfa* and *sbn* loci heavily restricts *S. aureus* virulence in a murine infection model (Beasley et al., 2011).

## Enterobactin

Enterobactin (**38**) is a siderophore most commonly associated with the gut microbiota, yet enterobactin is produced by the oral commensal *Rothia mucilaginosa* (Uranga et al., 2020) (Fig. 4f). The enterobactin BGC harbored by *R. mucilaginosa* was originally misidentified as a griseobactin BGC in a large-scale genome mining effort (Aleti et al., 2019). Arnow's assay indicated the presence of a catecholate siderophore, and a combination of MS/MS and NMR studies confirmed the siderophore was indeed enterobactin (Uranga et al., 2020).

The impact of enterobactin on other oral commensals suggests a previously unexplored ecological mechanism that shapes the oral community. *In vitro* studies suggest that the fitness effect of enterobactin in oral-residing *Streptococcus* spp. is uneven, necessitating future work to determine the role of enterobactin in a community context (Uranga et al., 2020). Interestingly, enterobactin-producing *R. mucilaginosa* may attenuate virulence in *S. aureus* by inhibiting staphyloxanthin production through a currently unknown mechanism (Uranga et al., 2020).

## Conclusions

The promise of the oral microbiota as a wellspring of novel natural products is supported by three major pillars. First, a bloom of genomic and metabolomic data suggests the oral microbiome harbors unexpected secondary metabolic diversity. Second, some oral natural products are known to play ecological roles with implications for oral microbial ecology, enabling study of the fundamental biology and ecology of the oral microbiota. Third, many oral natural products may influence therapeutic development. Antimicrobial and immune modulatory natural products are promising targets for further development. Siderophores might yield scaffolds for Trojan-horse antibiotics (Milner et al., 2013). Biosynthetic pathways for oral NPs involved in pathogenesis are exploitable targets for virulence attenuation; targeting such pathways to ablate virulence rather than kill pathogenic microbes outright might slow proliferation of antimicrobial resistance (Rasko and Sperandio, 2010). We anticipate exciting advances in genomics-driven natural products discovery from the nasal/oral microbiome and eagerly await deeper investigation of the role of small molecule natural products in nasal/oral microbial ecology.

## References

[bib1] Aiman S., Shehroz M., Munir M., Gul S., Shah M., Khan A. (2018). Species-wide genome mining of *P. putida* for potential secondary metabolites and drug-like natural products characterization. Journal of Proteomics & Bioinformatics, 11(1), 1–7.30618479

[bib2] Ajdić D., McShan W. M., McLaughlin R. E., Savić G., Chang J., Carson M. B., Primeaux C., Tian R., Kenton S., Jia H., Lin S., Qian T., Li S., Zhu H., Najar F., Lai H., White J., Roe B. A., Ferretti J. J. (2002). Genome sequence of *Streptococcus mutans* UA159, a cariogenic dental pathogen. Proceedings of the National Academy of Sciences of the USA, 99(22), 14434–14439. 12397186 10.1073/pnas.172501299 PMC137901

[bib3] Aleti G., Baker J. L., Tang X., Alvarez R., Dinis M., Tran N. C., Melnik A. V., Zhong C., Ernst M., Dorrestein P. C., Edlund A. (2019). Identification of the bacterial biosynthetic gene clusters of the oral microbiome illuminates the unexplored social language of bacteria during health and disease. mBio, 10, e00321–19. 30992349 10.1128/mBio.00321-19 PMC6469967

[bib4] Armstrong G. A. (1997). Genetics of eubacterial carotenoid biosynthesis: A colorful tale. Annual Review of Microbiology, 51, 629–659. 10.1146/annurev.micro.51.1.629 9343362

[bib5] Bangera M. G., Thomashow L. S. (1999). Identification and characterization of a gene cluster for synthesis of the polyketide antibiotic 2,4-diacetylphloroglucinol from *Pseudomonas fluorescens* Q2-87. Journal of Bacteriology, 181(10), 3155–3163. 10322017 10.1128/jb.181.10.3155-3163.1999 PMC93771

[bib6] Barbosa O. J., Rossoni R. D., Vilela S. F. G., de Alvarenga J. A., dos Santos Vellose M., de Azevedo Parata M. C., Jorge A. O. C., Junqueira J. C. (2016). *Streptococcus mutans* can modulate biofilm formation and attenuate the virulence of *Candida albicans*. PLoS One, 11(3), e0150457. 26934196 10.1371/journal.pone.0150457 PMC4774980

[bib7] Barry S. M., Challis G. L. (2009). Recent advances in siderophore biosynthesis. Current Opinion in Chemical Biology, 13(2), 205–215. 19369113 10.1016/j.cbpa.2009.03.008

[bib8] Beasley F. C., Marolda C. L., Cheung J., Buac S., Heinrichs D. E. (2011). *Staphylococcus aureus* transporters Hts, Sir, and Sst capture iron liberated from human transferrin by staphyloferrin A, staphyloferrin B, and catecholamine stress hormones, respectively, and contribute to virulence. Infection and Immunity, 79(6), 2345–2355. 21402762 10.1128/IAI.00117-11 PMC3125851

[bib9] Beasley F. C., Vines E. D., Grigg J. C., Zheng Q., Liu S., Lajoie G. A., Murphy M. E. P., Heinrichs D. E. (2009). Characterization of staphyloferrin A biosynthetic and transport mutants in *Staphylococcus aureus*. Molecular Microbiology, 72(4), 947–963. 19400778 10.1111/j.1365-2958.2009.06698.x

[bib10] Bitschar K., Sauer B., Focken J., Dehmer H., Moos S., Konnerth M., Schilling N. A., Grond S., Kalbacher H., Kurschus F. C., Götz F., Krismer B., Peschel A., Schittek B. (2019). Lugdunin amplifies innate immune responses in the skin in synergy with host- and microbiota-derived factors. Nature Communications, 10, 2730. 10.1038/s41467-019-10646-7 PMC6588697 31227691

[bib11] Blättner S., Das S., Paprotka K., Eilers U., Kirschke M., Kretschmer D., Remmele C. W., Dittrich M., Müller T., Schuelein-Voelk C., Hertlein T., Mueller M. J., Huettel B., Reinhardt R., Ohlsen K., Rudel T., Fraunholz M. J. (2016). *Staphylococcus aureus* exploits a non-ribosomal cyclic dipeptide to modulate survival within epithelial cells and phagocytes. PLoS Pathogens, 12(9), e1005857. 27632173 10.1371/journal.ppat.1005857 PMC5025175

[bib12] Böhme R., Jung G., Breitmaier E. (2005). Synthesis of the antibiotic (R)-reutericyclin via Dieckmann condensation. Helvetica Chimica Acta, 88, 2837–2841.

[bib13] Bowen W. H., Burne R. A., Wu H., Koo H. (2018). Oral biofilms: Pathogens, matrix, and polymicrobial interactions in microenvironments. Trends in Microbiology, 26(3), 229–242. 29097091 10.1016/j.tim.2017.09.008 PMC5834367

[bib14] Brown A. J. P., Odds F. C., Grow N. A. R. (2007). Infection-related gene expression in *Candida albicans*. Current Opinion in Microbiology, 10(4), 307–313. 17707687 10.1016/j.mib.2007.04.001

[bib15] Cagetti M. G., Mastroberardino S., Milia E., Cocco F., Lingström C. G. (2013). The use of probiotic strains in caries prevention: A systematic review. Nutrients, 5(7), 2530–2550. 23857225 10.3390/nu5072530 PMC3738986

[bib16] Carroll C. S., Moore M. M. (2018). Ironing out siderophore biosynthesis: A review of non-ribosomal peptide synthetase (NRPS)-independent siderophore synthases. Critical Reviews in Biochemistry and Molecular Biology, 53(4), 356–381. 29863423 10.1080/10409238.2018.1476449

[bib17] Cassat J. E., Skaar E. P. (2012). Metal ion acquisition in *Staphylococcus aureus*: overcoming nutritional immunity. Seminars in Immunopathology, 34, 215–235. 22048835 10.1007/s00281-011-0294-4 PMC3796439

[bib18] Chandra J. D., Kuhn D. M., Mukherjee P. K., Hoyer L. L., McCormick T., Ghannoum M. A. (2001). Biolfilm formation by the fungal pathogen *Candida albicans*: Development, architecture, and drug resistance. Journal of Bacteriology, 183, 5385–5394. 11514524 10.1128/JB.183.18.5385-5394.2001 PMC95423

[bib19] Chen L., Qin B., Du M., Zhong H., Xu Q., Li Y., Zhang P., Fan M. (2015). Extensive description and comparison of human supra-gingival microbiome in root caries and health. PLoS One, 10(2), e0117064. 25658087 10.1371/journal.pone.0117064 PMC4319720

[bib20] Chen T., Yu W., Izard J., Baranova O. V., Lakshmanan A., Dewhirst F. E. (2010). The Human Oral Microbiome Database: A web accessible resource for investigating oral microbe taxonomic and genomic information. Database, 2010, baq013. 20624719 10.1093/database/baq013 PMC2911848

[bib21] Cheung K., Beasley F. C., Liu S., Lajoie G. A., Heinrichs D. E. (2009). Molecular characterization of staphyloferrin B biosynthesis in *Staphylococcus aureus*. Molecular Microbiology, 74, 594–608. 19775248 10.1111/j.1365-2958.2009.06880.x

[bib22] Clauditz A., Resch A., Wieland K., Peschel A., Götz F. (2006). Staphyloxanthin plays a role in the fitness of *S. aureus* and its ability to cope with oxidative stress. Infection and Immunity, 74(8), 4950–4953. 16861688 10.1128/IAI.00204-06 PMC1539600

[bib23] Clifford J. C., Buchanan A., Vining O., Kidarsa T. A., Chang J. H., McPhail K. L., Loper J. E. (2016). Phloroglucinol functions as an intracellular and intercellular chemical messenger influencing gene expression in *Pseudomonas protegens*. Environmental Microbiology, 18(20), 3296–3308. 26337778 10.1111/1462-2920.13043

[bib24] Cotton J. L., Tao J., Balibar C. J. (2009). Identification and characterization of the *Staphylococcus aureus* gene cluster coding for staphyloferrin A. Biochemistry, 48, 1025–1035. 19138128 10.1021/bi801844c

[bib25] Courcol R. J., Trivier D., Bissinger M. C., Martin G. R., Brown M. R. W. (1997). Siderophore production by *Staphylococcus aureus* and identification of iron-regulated proteins. Infection and Immunity, 65(5), 1944–1948. 9125585 10.1128/iai.65.5.1944-1948.1997 PMC175248

[bib26] Dale S. E., Doherty-Kirby A., Lajoie G., Heinrichs D. E. (2004). Role of siderophore biosynthesis in virulence of *Staphylococcus aureus*: Identification and characterization of genes involved in production of a siderophore. Infection and Immunity, 72, 29–37. 14688077 10.1128/IAI.72.1.29-37.2004 PMC343950

[bib27] Dale S. E., Sebulsky M. T., Heinrichs D. E. (2004). Involvement of SirABC in iron-siderophore import in *Staphylococcus aureus*. Journal of Bacteriology, 186(24), 8356–8362. 15576785 10.1128/JB.186.24.8356-8362.2004 PMC532444

[bib28] Dewhirst F. E., Chen T., Izard J., Paster B. J., Tanner A. C. R., Yu W.-H., Lakshmanan A., Wade W. G. (2010). The human oral microbiome. Journal of Bacteriology, 192(19), 5002–5017. 20656903 10.1128/JB.00542-10 PMC2944498

[bib29] Ding T., Schloss P. D. (2014). Dynamics and associations of microbial community types across the human body. Nature, 509, 357–360. 24739969 10.1038/nature13178 PMC4139711

[bib30] Donia M. S., Fischbach M. A. (2015). Small molecules from the human microbiota. Science, 349(6246), 1254766. 26206939 10.1126/science.1254766 PMC4641445

[bib31] Drechsel H., Freund S., Nicholson G., Haag H., Jung O., Zahner H., Jung G. (1993). Purification and chemical characterization of staphyloferrin B, a hydrophilic siderophore from staphylococci. Biometals, 6, 185–192. 8400765 10.1007/BF00205858

[bib32] Edlund A., Garg N., Mohimani H., Gurevich A., He X., Shi W., Dorrestein P. C., McLean J. S. (2017). Metabolic fingerprints from the human oral microbiome reveal a vast knowledge gap of secreted small peptidic molecules. mSystems, 2(4), e00058–17. 28761934 10.1128/mSystems.00058-17 PMC5516222

[bib35] Gänzle M. G. (2004). Reutericyclin: biological activity, mode of action, and potential applications. Applied Microbiology and Biotechnology, 64, 326–332. 14735324 10.1007/s00253-003-1536-8

[bib36] Gänzle M. G., Hertel C., Hammes W. P. (1995). Antimicrobial activity in lactobacilli from sourdough. In Scheffers H. W. A., van Dijken J. P. (Eds.), Beijerinck Centennial Microbial physiology and gene regulation: emerging principles and applications (pp. 380–381). Delft University Press.

[bib33] Gänzle M. G., Höltzel A., Walter J., Jung G., Hammes W. P. (2000). Characterization of reutericyclin produced by *Lactobacillus reuteri* LTH2584. Applied and Environmental Microbiology, 66(10), 4325–4333. 11010877 10.1128/aem.66.10.4325-4333.2000 PMC92303

[bib34] Gänzle M. G., Vogel R. F. (2003). Contribution of reutericyclin production to the stable persistence of *Lactobacillus reuteri* in an industrial sourdough fermentation. International Journal of Food Microbiology, 80, 31–45. 12430769 10.1016/s0168-1605(02)00146-0

[bib37] Gao L., Xu T., Huang G., Jiang S., Gu Y., Chen F. (2018). Oral microbiomes: More and more importance in oral cavity and whole body. Protein Cell, 9, 488–500. 29736705 10.1007/s13238-018-0548-1 PMC5960472

[bib38] Grigg J. C., Cheung J., Heinrichs D. E., Murphy M. E. P. (2010). Specificity of staphyloferrin B recognition by the SirA receptor from *Staphylococcus aureus*. Journal of Biological Chemistry, 285(45), 34579–34588. 20810662 10.1074/jbc.M110.172924 PMC2966073

[bib39] Grow N. A. R., Brown A. J. P., Odds F. C. (2002). Fungal morphogenesis and host invasion. Current Opinion in Microbiology, 5(4), 366–371. 12160854 10.1016/s1369-5274(02)00338-7

[bib40] Gulick A. M. (2017). Nonribosomal peptide synthetase biosynthetic clusters of ESKAPE pathogens. Natural Product Reports, 34, 981–1009. 28642945 10.1039/c7np00029d PMC5551671

[bib41] Guo C. J., Chang F. Y., Wyche T. P., Backus K. M., Achker T. M., Funabashi M., Taketani M., Donia M. S., Nayfach S., Pollard K. S., Craik C. S., Cravatt B. F., Clardy J., Voigt C. A., Fischbach M. A. (2017). Discovery of reactive microbiota-derived metabolites that inhibit host proteases. Cell, 168(3), 517–526.e18. 28111075 10.1016/j.cell.2016.12.021 PMC5302092

[bib42] Gupta P., Sharma M., Arora N., Pruthi V., Poluri K. M. (2019). Chemistry and biology of farnesol and its derivatives: Quorum sensing molecules with immense therapeutic potential. Current Topics in Medicinal Chemistry, 18(22), 1937–1954. 10.2174/1568026619666181210124159 30526460

[bib43] Haag H., Fiedler H. P., Meiwes J., Drechsel H., Jung G., Zähner H. (1994). Isolation and biological characterization of staphyloferrin B, a compound with siderophore activity from staphylococci. FEMS Microbiol Letters, 115(2–3), 125–130. 10.1111/j.1574-6968.1994.tb06626.x 8138126

[bib44] Hahnel S., Rosentritt M., Bürgers R., Handel G. (2008). Adhesion of *Streptococcus mutans* NCTC 10449 to artificial teeth: An in vitro study. The Journal of Prosthetic Dentistry, 100, 309–315. 18922260 10.1016/S0022-3913(08)60212-7

[bib45] Hanses F., Roux C., Dunman P. M., Salzberger B., Lee J. C. (2014). *Staphylococcus aureus* gene expression in a rat model of infective endocarditis. Genome Medicine, 6, 93. 25392717 10.1186/s13073-014-0093-3 PMC4228149

[bib46] Hao T., Xie Z., Wang M., Liu L., Zhang Y., Wang W., Zhang Z., Zhao X., Li P., Guo Z., Gao S., Lou C., Zhang G., Merrit J., Horsman G. P., Chen Y. (2019). An anaerobic bacterium host system for heterologous expression of natural product biosynthetic gene clusters. Nature Communications, 10, 3665. 10.1038/s41467-019-11673-0 PMC6694145 31413323

[bib47] Hider R. C., Kong X. (2010). Chemistry and biology of siderophores. Natural Product Reports, 27, 637–657. 20376388 10.1039/b906679a

[bib48] Höltzel A., Gänzle M. G., Nicholson G. J., Hammes W. P., Jung G. (2000). The first low molecular weight antibiotic from lactic acid bacteria: Reutericyclin, a new tetramic acid. Angewandte Chemie, International Edition, 39(15), 2766–2768. 10934421

[bib49] Hong C. E., Jo S. H., Jo I. H., Jeong H., Park J. M. (2017). Draft genome sequence of the endophytic bacterium *Variovorax paradoxus* KB5, which has antagonistic activity against a phytopathogen, *Pseudomonas syringae* pv. *tomato* DC3000. Genome Announcements, 5(36), e00950–17. 28883145 10.1128/genomeA.00950-17 PMC5589539

[bib50] Hornby J. M., Jensen E. C., Lisec A. D., Tasto J. J., Jahnke B., Shoemaker R., Dussault P., Nickerson K. W. (2001). Quorum sensing in the dimorphic fungus *Candida albicans* is mediated by farnesol. Applied and Environmental Microbiology, 67(7), 2982–2992. 11425711 10.1128/AEM.67.7.2982-2992.2001 PMC92970

[bib51] Hornby J. M., Kebaara B. W., Nickerson K. W. (2003). Farnesol biosynthesis in *C. albicans*: Cellular response to sterol inhibition by zaragozic acid B. Antimicrobial Agents Chemotherapy, 47(7), 2366–2369. 12821501 10.1128/AAC.47.7.2366-2369.2003 PMC161837

[bib52] Hu Z., Zhang W. (2020). Signaling natural products from human pathogenic bacteria. ACS Infectious Diseases, 6(1), 25–33. 31617342 10.1021/acsinfecdis.9b00286 PMC7609248

[bib53] Ibrahim A., Yang L., Johnston C., Liu X., Ma B., Magarvey N. A. (2012). Dereplicating nonribosomal peptides using an informatic search algorithm for natural products (iSNAP) discovery. Proceedings of the National Academy of Sciences of the USA, 109(47), 19196–19201. 23132949 10.1073/pnas.1206376109 PMC3511102

[bib54] Jakubovics N. S., Yassin S. A., Rickard A. H. (2014). Chapter Two—Community interactions of oral streptococci. In Sariaslani S., Gadd G. M. (Eds.), Advances in Applied Microbiology. Academic Press. 10.1016/B978-0-12-800261-2.00002-5 24581389

[bib55] Jia G., Zhi A., Lai P. F. H., Wang G., Xia Y., Xiong Z., Zhang H., Che B., Ai L. (2018). The oral microbiota—A mechanistic role for systemic diseases. British Dental Journal, 224, 447–455. 29569607 10.1038/sj.bdj.2018.217

[bib56] Johnston C. W., Wyatt M. A., Li X., Ibrahim A., Shuster J., Southam G., Magarvey N. A. (2013). Gold biomineralization by a metallophore from a gold-associated microbe. Nature Chemical Biology, 9, 241–243. 23377039 10.1038/nchembio.1179

[bib57] Joyner P. M., Liu J., Zhang Z., Meritt J., Qi F., Cichewicz R. H. (2010). Mutanobactin A from the human oral pathogen *Streptococcus mutans* is a cross-kingdom regulator of the yeast-mycelium transition. Organic & Biomolecular Chemistry, 8, 5486–5489. 20852771 10.1039/c0ob00579g PMC2992086

[bib58] Kawamura I., Yagi T., Hatakeyama K., Ohkura T., Ohkusu K., Takahashi Y., Kojima S., Hasegawa Y. (2011). Recurrent vascular catheter-related bacteremia caused by *Delftia acidocorans* with different antimicrobial susceptibility profiles. Journal of Infection and Chemotherapy, 17, 111–113. 20628778 10.1007/s10156-010-0089-x

[bib59] Klapper M., Paschold A., Zhang S., Weigel C., Dahse H., Götze S., Pace S., König S., Rao Z., Reimer L., Werz O., Stallforth P. (2019). Bioactivity and mode of action of bacterial tetramic acids. ACS Chemical Biology, 14, 1693–1697. 31294961 10.1021/acschembio.9b00388

[bib60] Konetschny-Rapp S., Jung G., Meiwes J., Zahner H. (1990). Staphyloferrin A: A structurally new siderophore from staphylococci. European Journal of Biochemistry FEBS, 191, 65–74. 10.1111/j.1432-1033.1990.tb19094.x 2379505

[bib62] Krismer B., Weidenmaier C., Zipperer A., Peschel A. (2017). The commensal lifestyle of *Staphylococcus aureus* and its interactions with the nasal microbiota. Nature Reviews Microbiology, 15, 675–687. 29021598 10.1038/nrmicro.2017.104

[bib63] Kuramitsu H. K., He X., Lux R., Andreson M. H., Shi W. (2007). Interspecies interaction within oral microbial communities. Microbiology and Molecular Biology Reviews, 71, 653–670. 18063722 10.1128/MMBR.00024-07 PMC2168648

[bib64] Laakso H. A., Marolda C. L., Pinter T. B., Stillman M. J., Heinrichs D. E. (2016). A heme-responsive regulator controls synthesis of staphyloferrin B in *Staphylococcus aureus*. Journal of Biological Chemistry, 291(1), 29–40. 26534960 10.1074/jbc.M115.696625 PMC4697164

[bib65] Lamont R. J., Koo H., Hajishengallis G. (2018). The oral microbiota: Dynamic communities and host interactions. Nature Reviews Microbiology, 16, 745–759. 30301974 10.1038/s41579-018-0089-x PMC6278837

[bib66] Laux C., Peschel A., Krismer B. (2019). Chapter 45: *Staphylococcus aureus* colonization of the human nose and interactions with other microbiome members. In Fischetti V. A., Novick R. P., Ferretti J. J., Portnoy D. A., Braunstein M., Rood J. I. (Eds.), Gram-positive pathogens. American Society for Microbiology Press. 10.1128/microbiolspec.gpp3-0029-2018 PMC11590430 31004422

[bib67] Lee J. H., Cho H. S., Kim Y., Kim J. A., Banskota S., Cho M. H., Lee J. (2013). Indole and 7-benzyloxyindole attenuate the virulence of *Staphylococcus aureus*. Applied Microbiology and Biotechnology, 97, 4543–4553. 23318836 10.1007/s00253-012-4674-z

[bib68] Leejae S., Hasap L., Voravuthikunchai S. P. (2013). Inhibition of staphyloxanthin biosynthesis in *Staphylococcus aureus* by rhodomyrtone, a novel antibiotic candidate. Journal of Medical Microbiology, 62(3), 421–428. 23242641 10.1099/jmm.0.047316-0

[bib69] Lemos J. A., Palmer S. R., Zeng L., Wen Z. T., Kajfasz J. K., Freires I. A., Abranches J. A., Brady L. J. (2019). Chapter 27: The biology of *Streptococcus mutans*. In Fischetti V. A., Novick R. P., Ferretti J. J., Portnoy D. A., Braunstein M., Rood J. I. (Eds.), Gram-positive pathogens. American Society for Microbiology Press. 10.1128/microbiolspec.gpp3-0051-2018 PMC6615571 30657107

[bib70] Li Z.-R., Du Y., Sun J., Pan A., Zeng L., Maboudian R., Burne R. A., Qian P.-Y., Zhang W. (In press). Mutanofaction promotes bacteria adhesion and biofilm formation of cariogenic Streptococcus mutans. Nature Chemical Biology. Retrieved from https://www.biorxiv.org/content/10.1101/2020.08.22.262196v1.abstract. 10.1038/s41589-021-00745-2 33664521

[bib71] Lin X. B., Lohans C. T., Duar R., Zheng J., Vederas J. C., Walter J., Gänzle M. G. (2015). Genetic determinants of reutericyclin biosynthesis in *Lactobacillus reuteri*. Applied and Environmental Microbiology, 81(6), 2032–2041. 25576609 10.1128/AEM.03691-14 PMC4345372

[bib72] Lindsay A. K., Deveau A., Piispanen A. E., Hogan D. A. (2012). Farnesol and cyclic AMP signaling effects on the hypha-to-yeast transition in *C. albicans*. Eukaryotic Cell, 11(10), 1219–1225. 22886999 10.1128/EC.00144-12 PMC3485915

[bib73] Liu C. I., Liu G. Y., Song Y., Yin F., Hensler M. E., Jeng W. Y., Nizet V., Wang A. H. J., Oldfield E. (2008). A cholesterol biosynthesis inhibitor blocks *Staphylococcus aureus* virulence. Science, 319(5868), 1391–1394. 18276850 10.1126/science.1153018 PMC2747771

[bib74] Liu G. Y., Essex A., Buchanan J. T., Datta V., Hoffman H. M., Bastian J. F., Fierer J., Nizet V. (2005). *Staphylococcus aureus* golden pigment impairs neutrophil killing and promotes virulence through its antioxidant activity. Journal of Experimental Medicine, 202, 209–215. 16009720 10.1084/jem.20050846 PMC2213009

[bib75] Liu L., Hao T., Xie Z., Horsman G. P., Chen Y. (2016). Genome mining unveils widespread natural product biosynthetic capacity in human oral microbe *Streptococcus mutans*. Scientific Reports, 6, 37479. 27869143 10.1038/srep37479 PMC5116633

[bib76] Lloyd-Price J., Mahurkar A., Rahnavard G., Crabtree J., Orvis J., Hall A. B., Brady A., Creasy H. H., McCracken C., Giglio M. G., McDonald D., Franzosa E. A., Knight R., White O., Huttenhower C. (2017). Strains, functions, and dynamics in the expanded Human Microbiome Project. Nature, 550, 61–66. 28953883 10.1038/nature23889 PMC5831082

[bib77] Loesche W. J. (1986). Role of *Streptococcus mutans* in human dental decay. Microbiological Reviews, 50(4), 353–380. 3540569 10.1128/mr.50.4.353-380.1986 PMC373078

[bib78] Mahmood S., Taylor K. E., Overman T. L., McCormick M. I. (2012). Acute infective endocarditis caused by Delftia acidovorans, a rare pathogen complication intravenous drug use. Journal of Clinical Microbiology, 50(11), 3799–3800. 22933597 10.1128/JCM.00553-12 PMC3486206

[bib79] Maura D., Ballok A. E., Rahme L. G. (2016). Consideration and caveats in anti-virulence drug development. Current Opinion in Microbiology, 33, 41–46. 27318551 10.1016/j.mib.2016.06.001 PMC5069136

[bib80] Meiwes J., Fiedler H.-P., Haag H., Zähner H., Konetschny-Rapp S., Jung G. (1990). Isolation and characterization of staphyloferrin A, a compound with siderophore activity from *Staphylococcus hyicus* DSM 20549. FEMS Microbiology Letters, 67(1–2), 201–205. 10.1111/j.1574-6968.1990.tb13863.x 2139423

[bib81] Merritt J., Qi F. (2012). The mutacins of *Streptococcus mutans*: Regulation and ecology. Molecular Oral Microbiology, 27(2), 57–69. 22394465 10.1111/j.2041-1014.2011.00634.x PMC3296966

[bib82] Miethke M., Marahiel M. A. (2007). Siderophore-based iron acquisition and pathogen control. Microbiology and Molecular Biology Reviews, 71, 413–451. 17804665 10.1128/MMBR.00012-07 PMC2168645

[bib83] Milner S. J., Seve A., Snelling A. M., Thomas G. H., Kerr K. G., Routledge A., Duhme-Klair A. K. (2013). Staphyloferrin A as siderophore-component in fluoroquinolone-based Trojan horse antibiotics. Organic & Biomolecular Chemistry, 11, 3461–3468. 23575952 10.1039/c3ob40162f

[bib84] Milshteyn A., Colosimo D. A., Brady S. F. (2018). Accessing bioactive natural products from the human microbiome. Cell Host & Microbe, 23(6), 725–736. 29902438 10.1016/j.chom.2018.05.013 PMC7232905

[bib85] Mousa W. K., Athar B., Merwin N. J., Magarvey N. A. (2017). Antibiotics and specialized metabolites from the human microbiota. Natural Product Reports, 34, 1302–1331. 29018846 10.1039/c7np00021a

[bib86] Mutaguchi Y., Ohmori T., Akano H., Doi K., Ohshima T. (2013). Distribution of D-amino acids in vinegars and involvement of lactic acid bacteria in the production of D-amino acids. SpringerPlus, 2, 691. 24422181 10.1186/2193-1801-2-691 PMC3884085

[bib87] Mutaguchi Y., Ohmori T., Wakamatsu T., Doi K., Ohshima T. (2013). Identification, purification, and characterization of a novel amino acid racemase, isoleucine 2-epimerase, from *Lactobacillus* species. Journal of Bacteriology, 195, 5207–5215. 24039265 10.1128/JB.00709-13 PMC3811583

[bib88] Navaranthna D., Hornby J. M., Krishnan N., Parkhurst A., Duhamel G. E., Nickerson K. W. (2007). Effect of farnesol on a mouse model of systemic candidiasis, determined by use of a DPP3 knockout mutant of *Candida albicans*. Infection and Immunity, 75(4), 1609–1618. 17283095 10.1128/IAI.01182-06 PMC1865729

[bib89] Park R. Y., Sun H. Y., Choi M. H., Bai Y. H., Shin S. H. S. (2005). *Staphylococcus aureus* siderophore-mediated iron-acquisition system plays a dominant and essential role in the utilization of transferrin-bound iron. Journal of Microbiology (Seoul, Korea), 43(2), 183–190. 15880095

[bib90] Pelz A., Wieland K., Putzbach K., Hentschel P., Albert K., Götz F. (2005). Structure and biosynthesis of staphyloxanthin from *Staphylococcus aureus*. Journal of Biological Chemistry, 280, 32493–32498. 16020541 10.1074/jbc.M505070200

[bib91] Polke M., Leonhardt I., Kurzai O., Jacobsen I. D. (2018). Farnesol signaling in *Candida albicans*—More than just communication. Critical Reviews in Microbiology, 44(2), 230–243. 28609183 10.1080/1040841X.2017.1337711

[bib92] Proctor D. M., Relman D. A. (2017). The landscape ecology and microbiota of the human nose, mouth, and throat. Cell Host & Microbe, 21(4), 421–432. 28407480 10.1016/j.chom.2017.03.011 PMC5538306

[bib93] Ramage G., Saville S. P., Wickes B. L., Lopez-Ribot J. L. (2002). Inhibition of *Candida albicans* biofilm formation by farnesol, a quorum-sensing molecule. Applied and Environmental Microbiology, 53(6), 2392–2401. 10.1128/AEM.68.11.5459-5463.2002 PMC129887 12406738

[bib94] Rasko D. A., Sperandio V. (2010). Anti-virulence strategies to combat bacteria-mediated disease. Nature Reviews Drug Discovery, 9, 117–128. 20081869 10.1038/nrd3013

[bib95] Reith F., Fairbrother L., Nolze G., Wilhelmi O., Clode P. L., Gregg A., Parsons J. E., Wakelin S. A., Pring A., Hough R., Southam G., Brugger J. (2010). Nanoparticle factories: Biofilms hold the key to gold dispersion and nugget formation. Geology, 38(9), 843–846.

[bib96] Ribeiro L., Fumagalli F., Mello R. B., Froes T. Q., da Silva M. V. S., Villamizar Gomez S. M., Barros T. F., Emery F. S., Castilho M. S. (2020). Structure–activity relationships and mechanism of action of tetragomycin derivatives as inhibitors of *Staphylococcus aureus* staphyloxanthin biosynthesis. Microbial Pathogenesis, 144, 104127. 32169485 10.1016/j.micpath.2020.104127

[bib97] Sakr A., Bregeon F., Mege J.-L., Rolain J.-M., Blin O. (2018). *Staphylococcus aureus* nasal colonization: An update on mechanisms, epidemiology, risk factors, and subsequent infections. Frontiers in Microbiology, 9(9), 2419. 30349525 10.3389/fmicb.2018.02419 PMC6186810

[bib98] Saville S. P., Lazzell A. L., Monteagudo C., Lopez-Ribot J. L. (2003). Engineered control of cell morphology in vivo reveals distinct roles for yeast and filamentous forms of *Candida albicans* during infection. Eukaryotic Cell, 2(5), 1053–1060. 14555488 10.1128/EC.2.5.1053-1060.2003 PMC219382

[bib99] Schilling N. A., Berscheid A., Schumacher J., Saur J. S., Konnerth M. C., Wirtz S. N., Beltran-Belena J. M., Zipperer A., Krismer B., Peschel A., Kalbacher H., Brötz-Oesterhelt H., Steinem C., Grond S. (2019). Synthetic lugdunin analogues reveal essential structural motifs for antimicrobial action and proton translocation capability. Angewandte Chemie, International Edition, 58(27), 9234–9238. 31059155 10.1002/anie.201901589 PMC6618241

[bib100] Secor P. R., Jennings L. K., James G. A., Kirker K. R., Pulcini E. D., McInnerney K., Gerlach R., Livinghouse T., Hilmer J. K., Bothner B., Fleckman P., Olerud J. E., Stewart P. S. (2012). Phevalin (aureusimine B) production by *Staphylococcus aureus* biofilm and impacts on human keratinocyte gene expression. PLoS One, 7(7), e40973. 22808288 10.1371/journal.pone.0040973 PMC3396627

[bib101] Song Y., Lin F., Yin F., Hensler M., Rodrigues Poveda C. A., Mukkamala D., Cao R., Wang H., Morita C. T., Gonzalez Pacanowska D., Nizet V., Oldfield E. (2009). Phosphonosulfonates are potent, selective inhibitors of dehydrosqualene synthase and staphyloxanthin biosynthesis in *Staphylococcus aureus*. Journal of Medicinal Chemistry, 52(4), 976–988. 19191557 10.1021/jm801023u PMC2765255

[bib102] Song Y., Liu C., Lin F., No J. H., Hensler M., Liu Y. L., Jeng W. Y., Low J., Liu G. Y., Nizet V., Wang A. H. J., Oldfield E. (2009). Inhibition of staphyloxanthin virulence factor biosynthesis in *Staphylococcus aureus*: In vitro, in vivo, and crystallographic results. Journal of Medicinal Chemistry, 52(13), 3869–3880. 19456099 10.1021/jm9001764 PMC2753857

[bib103] Speziali C. D., Dale S. E., Henderson J. A., Vines E. D., Heinrichs D. E. (2006). Requirement of *Staphylococcus aureus* ATP-binding cassette-ATPase FhuC for iron-restricted growth and evidence that it functions with more than one iron transporter. Journal of Bacteriology, 188(6), 2048–2055. 16513734 10.1128/JB.188.6.2048-2055.2006 PMC1428144

[bib104] Sun F., Cho H., Jeong D. W., Li C., He C., Bae T. (2010). Aureusimines in *Staphylococcus aureus* are not involved in virulence. PLoS One, 5, e15703. 21209955 10.1371/journal.pone.0015703 PMC3012096

[bib105] Tang X., Kudo Y., Baker J. L., LaBonte S., Jordan P. A., McKinnie S. M. K., Guo J., Huan T., BS M., Edlund A. (2020). Cariogenic S. mutans produces tetramic acid strain-specific antibiotics that impair commensal colonization. ACS Infectious Diseases, 6, 563–571. 31906623 10.1021/acsinfecdis.9b00365 PMC7150634

[bib106] Tejman-Yarden N., Robinson A., Davidov Y., Shulman A., Varvak A., Reyes F., Rahav G., Nissan I. (2019). Delftibactin-A, a non-ribosomal peptide with broad antimicrobial activity. Frontiers in Microbiology, 10, 2377. 31681234 10.3389/fmicb.2019.02377 PMC6808179

[bib107] Uranga C. C., Arroyo P., Buggan B. M., Gerwick W. H., Edlund A. (2020). Commensal oral Rothia mucilaginosa produces enterobactin, a metal-chelating siderophore. mSystems, 5, e00161–20. 32345739 10.1128/mSystems.00161-20 PMC7190385

[bib108] Wall G., Montelong-Jauregui D., Bonifacio B. V., Lopez-Ribot J. L., Uppuluri P. (2019). *Candida albicans* biofilm growth and dispersal: contributions to pathogenesis. Current Opinion in Microbiology, 52, 1–6. 31085405 10.1016/j.mib.2019.04.001 PMC6842673

[bib109] Wang M., Xie Z., Tang S., Chang E. L., Tang Y., Guo Z., Cui Y., Wu B., Ye T., Chen Y. (2020). Reductase of mutanobactin synthetase triggers sequential C–C macrocyclization, C–S bond formation, and C–C bond cleavage. Organic Letters, 22, 960–964. 31917593 10.1021/acs.orglett.9b04501

[bib110] Wang X., Du L., You J., King J. B., Cichewicz R. H. (2012). Fungal biofilm inhibitors from a human oral microbiome-derived bacterium. Organic & Biomolecular Chemistry, 10, 2044–2050. 22281750 10.1039/c2ob06856g

[bib111] Wertheim H. F. L., Melles D. C., Vos M. C., van Leeuwen W., van Belkum A., Verbrugh H. A., Nouwen J. L. (2005). The role of nasal carriage in *Staphylococcus aureus* infections. The Lancet Infectious Diseases, 5(12), 751–762. 16310147 10.1016/S1473-3099(05)70295-4

[bib112] Wilson D. J., Shi C., Teitelbaum A. M., Gulick A. M., Aldrich C. C. (2013). Characterization of AusA: A dimodular nonribosomal peptide synthetase responsible for the production of aureusimine pyrazinones. Biochemistry, 52(5), 926–937. 23302043 10.1021/bi301330q PMC3577359

[bib113] Wu C., Cichewicz R., Li Y., Liu J., Roe B., Ferretti J., Merritt J., Qi F. (2010). Genomic Island TnSmu2 of *Streptococcus mutans* harbors a nonribosomal peptide synthetase-polyketide synthase gene cluster responsible for the biosynthesis of pigments involved in oxygen and H_2_O_2_ tolerance. Applied and Environmental Microbiology, 76(17), 5815–5826. 20639370 10.1128/AEM.03079-09 PMC2935078

[bib114] Wyatt M. A., Mok M. C. Y., Junop M., Magarvey N. A. (2012). Heterologous expression and structural characterization of a pyrazinone natural product assembly line. Chembiochem, 13, 2408–2415. 23070851 10.1002/cbic.201200340

[bib116] Wyatt M. A., Wang W., Roux C. M., Beasly F. C., Heinrichs D. E., Dunman P. M., Magarvey N. A. (2010). *Staphylococcus aureus* nonribosomal peptide secondary metabolites regulate virulence. Science, 329, 294–296. 20522739 10.1126/science.1188888

[bib115] Wyatt M. A., Wang W., Roux C. M., Beasley F. C., Heinrichs D. E., Dunman P. M., Magarvey N. A. (2011). Clarification of “*Staphylococcus aureus* nonribosomal peptide secondary metabolites regulate virulence.” Science, 333, 1381. 20522739 10.1126/science.1188888

[bib117] Yang Y., Zhao X., Le M. H. A., Zijlstra R. T., Gänzle M. G. (2015). Reutericyclin producing *Lactobacillus reuteri* modulates development of fecal microbiota in weanling pigs. Frontiers in Microbiology, 6, 762. 26284047 10.3389/fmicb.2015.00762 PMC4516970

[bib118] Zhao X., Wang W., Blaine A., Kane S. T., Zijlstra R. T., Gänzle M. G. (2019). Impact of probiotic *Lactobacillus* sp. on autochthonous lactobacilli in weaned piglets. Journal of Applied Microbiology, 126, 242–254. 30276941 10.1111/jam.14119

[bib119] Zheng J., Gänzle M. G., Lin C. B., Ruan L., Sun M. (2015). Diversity and dynamics of bacteriocins from human microbiome. Environmental Microbiology, 17(6), 2133–2143. 25346017 10.1111/1462-2920.12662

[bib120] Zimmermann M., Fischbach M. A. (2010). A family of pyrazinone natural products from a conserved nonribosomal peptide synthetase in *Staphylococcus aureus*. Chemistry & Biology, 17(9), 925–930. 20851341 10.1016/j.chembiol.2010.08.006

[bib121] Zipperer A., Konnerth M. C., Laux C., Berscheid A., Janek D., Weidenmaier C., Burian M., Schilling N. A., Slavetinsky C., Marschal M., Willmann M., Kalbacher H., Schittek B., Brötz-Oesterhelt H., Grond S., Pescehl A., Krismer B. (2016). Human commensals producing a novel antibiotic impair pathogen colonization. Nature, 535, 511–516. 27466123 10.1038/nature18634

[bib122] Zvanych R., Lukenda N., Li X., Kim J. J., Tharmarajah S., Magarvey N. A. (2015). Systems biosynthesis of secondary metabolic pathways within the oral human microbiome member *Streptococcus mutans*. Molecular Biosystems, 11, 97–104. 25209237 10.1039/c4mb00406j

